# Functional retroperitoneal paraganglioma invading the inferior vena cava in the elderly, a case report and literature review

**DOI:** 10.1016/j.ijscr.2023.108547

**Published:** 2023-07-21

**Authors:** Mohamed Aymane Loukili, Imane Assarrar, Nada El Yamani, Anass Haloui, Siham Rouf, Hanane Latrech

**Affiliations:** a Department of Endocrinology-Diabetology and Nutrition, Mohammed VI University Hospital Center, Faculty of Medicine and Pharmacy, University of Mohammed 1st, Oujda, Morocco; b Laboratory of Epidemiology, Clinical Research and Public Health, Faculty of Medicine and Pharmacy, University of Mohammed first, Oujda, Morocco; c Department of Anatomical Pathology, Mohammed VI University Hospital Center, Faculty of Medicine and Pharmacy, University of Mohammed 1st, Oujda, Morocco

**Keywords:** Case report, Paraganglioma, Retroperitoneal, Functional

## Abstract

**Introduction and importance:**

Phaeochromocytomas and paragangliomas are rare neuroendocrine neoplasms that grow outside the adrenal gland and arise from the primitive neural crest cells. The retroperitoneal location is extremely rare with an incidence of 2–8 per million.

**Case presentation:**

Here we report a case of an 80 years old man presenting with abdominal pain and vomiting associated with hypertensive peaks and weight loss. CT scan showed a retroperitoneal para-aortic tumor invading the inferior vena cava, with significantly elevated urinary catecholamine levels. Histopathological and immunohistochemistry examinations confirmed the diagnosis of paraganglioma. A medical preparation by alpha-blockers was performed. Complete resection of the tumor with the reconstruction of the vena cava was achieved without postoperative complications. After surgery, blood pressure and HbA1c were on the targets and the urinary catecholamine levels were normal.

**Clinical discussion:**

The diagnosis of paragangliomas is suspected by clinical symptoms in the case of functional paragangliomas and the confirmation is biological by the plasmatic or urinary catecholamines. Non-functional paragangliomas often represent a diagnostic challenge. In our case, the large size, the location of the tumor, and the invasion of adjacent structures represented a surgical challenge to perform a complete resection.

**Conclusion:**

In the elderly, this pathology is quite uncommon. Retroperitoneal paraganglioma is a rare location of this type of tumor. Endocrinologists, surgeons, and anesthesiologists should work together to ensure an appropriate diagnosis and treatment of paraganglioma. The gold standard treatment is the complete resection after a medical preparation.

## Highlights

• Retroperitoneal paragalglioma is a rare location of this type of tumor and causes a diagnostic challenge.
• Paraganglioma may occur at any age, frequently in the fourth decade. Its occurrence in the elderly is exceptional.
• In our case, a reconstruction of the inferior vena cava was performed using prosthetic material.
• It constituted a surgical challenge due to the localization, the hemorrhagic risk, and the increased volume of the tumor.

## 1 Introduction

Pheochromocytomas and paragangliomas (PGLs) are neuroendocrine tumors composed of chromaffin cells arising from dispersed specialized neural crest cells [1]. PGLs are situated outside of the adrenal glands. It occurs frequently in the fourth decade [2]. The retroperitoneal location is extremely rare with an incidence of 2–8 per million [3]. The thoracic and abdominal tumors produce excessive catecholamines in 85 % of cases. However, PGLs localized in the base of the skull (carotid, vagal, tympanic, or jugular glomus) are non-functional in 90 % of cases [2]. Non-functional PGL often represents a diagnostic challenge due to the absence of symptoms related to the lack of secretion of catecholamines [4]. They are frequently locally invasive and associated with a high incidence of local recurrence [5].

In this paper, we report a case of a giant retroperitoneal functional PGL invading the inferior vena cava (IVC) in an elderly man 80 years old which is a rare condition at this age. This case constituted a diagnostic challenge due to the delayed onset of the symptoms and a surgical challenge due to the localization, the hemorrhagic risk, the anesthetic risk, and the increased volume of the tumor. This case has been reported in line with the SCARE criteria [6].

## 2 Case presentation

We report the case of an 80 years-old man who presented to the Department of Endocrinology-Diabetology and Nutrition in 2020 with epigastric pain, associated with postprandial vomiting, and important weight loss. The patient also reported Menard triad approximately 2 months before admission. The patient had no previous medical history. The patient also presented hypertensive peaks and was on Amlodipine medication in the last 2 months. Clinical examination did not reveal any significant abnormality. Biochemical parameters revealed diabetes mellitus, probably secondary to the PGL, that required insulin treatment.

**Fig. 1:**
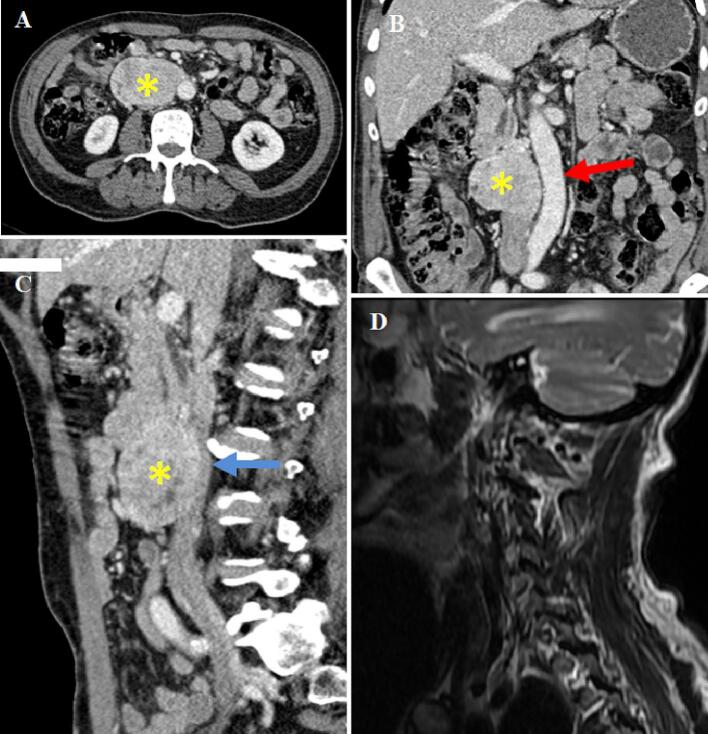
Imaging findings: contrast CT: an axial image of the portal venous phase (A), coronal image (B), sagittal image (C), the retroperitoneal mass of 8×7×3 cm with homogeneous aspect and hemorrhagic remodeling (*), backflowing the abdominal aorta (red arrow), and compressing the IVC (blue arrow); T2 weighted sagittal MRI image of the skull and the neck showing the absence of metastasis in this stage (D). (For interpretation of the references to color in this figure legend, the reader is referred to the web version of this article.)

Abdominal sonography and ultrasound endoscopy revealed a heterogeneous hyperechoic mass attached to the descending portion of the duodenum and aorta. The CT scan of the chest, abdomen, and pelvis (Fig. 1) showed a right retroperitoneal para-aortic process with regular and well-defined borders, rising early and heterogeneously after contrast injection, measuring 72 × 57 mm and extending over 69 mm. It represses and laminates the IVC, which remains permeable (Fig. 1). The adrenal glands were normal.

Urinary catecholamine levels were significantly elevated; Metanephrine (MN) at 59,13 μmol/24 h (normal value (NV)):<2,5 μmol/24 h, 23× the upper normal limit, Liquid Chromatography coupled to tandem Mass Spectrometry (LC-MS/MS) and normetanephrine (NMN) at 46 μmol/24 h (NV: <1,5 μmol/24 h, 30× the upper normal limit, LC-MS/MS). MRI did not show other localization in the neck and base of the skull (Fig. 1).

**Fig. 2:**
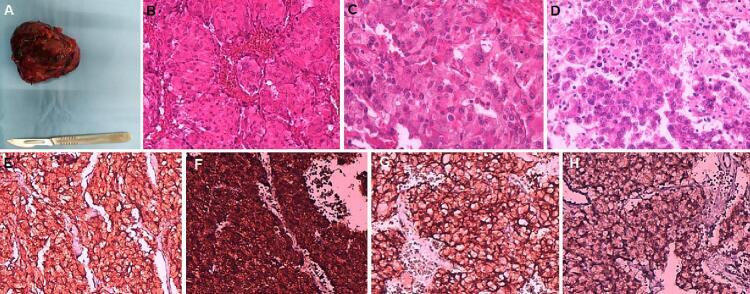
The excised tumor (80×70×30 mm) (A). Histopathological examination showing nesting (“Zellballen” architecture) and trabecular pattern of tumoral cells within a prominent vascular network (×40) (B). Tumor nests were surrounded by sustentacular cells (×40) (C). Tumor cells were large, provided with a rounded nucleus, fine chromatin, inconspicuous nucleoli, and abundant, slightly eosinophilic cytoplasm (×40) (D). Immunohistochemistry staining showing diffuse expression of Synaptophysin (×40) (E), chromogranin (×40) (F), and CD56 (×40) (G). Sustentacular cells showing expression of PS100 (×40) (H).

Surgery was performed after 2 weeks of α-adrenergic receptor blockage by doxazosin at the dose of 4 mg/24 h. After the stabilization of the blood pressure, the patient was transferred to the Surgical Oncology Department. The tumor was resected using a laparotomy by upper midline incision was made given the large size of the tumor. Intraoperatively, a retroperitoneal mass was found abutting the pancreatic uncus and the third portion of the duodenum. It also infiltrated the infrarenal IVC. Heparinized saline was used during the procedure to prevent vein thrombosis. Initially, surgeons clamped the IVC to perform reconstruction and adrenal and kidney veins to prevent catecholaminergic discharge. A complete resection of the mass (Fig. 2) with circumferential resection of the infrarenal IVC was performed and a polytetrafluoroethylene (PTFE) graft was used to replace the IVC. To avoid a bifurcated reconstruction, tailoring of the graft with the remaining IVC was completed. The distal anastomosis was achieved end-to-end under the renal veins. Vascular reconstruction was performed by vascular surgeons.

Doxazosin was replaced after surgery by Calcium Channel Blockers for three days, then the patient was weaned from antihypertensive treatment. Blood pressure monitoring was normal. The postoperative course was uneventful. Insulin therapy was stopped one week later considering the optimal glycemic control obtained after surgery.

The histopathological examination confirmed the diagnosis and showed Nesting (“Zellballen” architecture) and trabecular pattern of tumoral cells within a prominent vascular network. Tumor nests were surrounded by sustentacular cells. Tumor cells were large, provided with a rounded nucleus, fine chromatin, inconspicuous nucleoli, and abundant, slightly eosinophilic cytoplasm. Immunohistochemistry staining showed diffuse expression of Synaptophysin, chromogranin, CD56, and sustentacular cells showed expression of PS100 (Fig. 2).

Urinary catecholamine levels were normal: Metanephrine was at 0,98 nmol/l (NV:<2,5 μmol/24 h, LC-MS/MS), and Normetanephrine was at 4,01 nmol/l (NV: <1,5 μmol/24 h, LC-MS/MS) two weeks after surgery. At the 3-month follow-up, blood pressure was on target and HbA1c was 5,8 % without treatment. Given the moderate elevation of normetanephrine at 1,5× the upper normal limit, urinary catecholamine levels were assessed 6 months later and were normal. The patients didn't exhibit any sign of recurrence during 3 years of follow-up until 2023.

## 3 Discussion

PGL is a rare neuroendocrine tumor with a neuroectodermal origin, arising from sympathetic and parasympathetic paraganglia. Neuroendocrine tumors are classified as chromaffin cell tumors, most often functional releasing catecholamines (including adrenal form: pheochromocytoma which represents 80 %, and extra-adrenal forms: thoracic or abdominal PGL, which represents 20 %), and non-chromaffin cells tumors, rarely functional (including cervical PGL) [7]. Intra-abdominal PGL is known as the most common site of PGL, usually in the organ of Zuckerkandl at the aortic bifurcation. Our patient had a retroperitoneal para-aortic tumor invading the IVC, this location is rarely reported in the literature [8].

PGL may occur at any age, frequently in the fourth decade. Its occurrence in the elderly is exceptional. Our case was asymptomatic and exhibited clinical symptoms at the age of 80 years. There is no gender predominance [2]. It's a very rare disease; the annual incidence of PGLs has been estimated to be around 0.6 per 100,000 people [9]. The prevalence of genetic forms of PGLs is estimated at 40 %, including Von Hippel-Lindau syndrome, multiple endocrine neoplasia type 2, neurofibromatosis type 1, the Carney triad, and gene mutations of the subunits of succinate dehydrogenase [2].

Catecholamines are synthesized from tyrosine in the chromaffin cells of the adrenal medulla and the central and peripheral sympathetic neurons. Tyrosine is converted to dopamine by tyrosine hydroxylase, which is then converted to norepinephrine by dopamine hydroxylase. Norepinephrine is converted in part to adrenaline by phenylethanolamine-*N*-methyltransferase. The adrenal medulla produces adrenaline (80 % of secretion) and norepinephrine (20 % of secretion); while sympathetic neurons synthesize only norepinephrine [10].

The clinical manifestations of PGL are variable, they can be related either to the hypersecretion of catecholamines, to the tumor syndrome due to the mass effect exerted by the tumor on the neighboring organs, or to both simultaneously. Hypertension is the most common sign, most often permanent, sometimes paroxysmal. It is associated with tachycardia, mainly sinus tachycardia as an effect of catecholamines on vascular alpha-adrenergic receptors. An unusual hypertensive flare-up during surgery should evoke the diagnosis of PGL. More rarely, hypotension related to dopamine co-secretion may be found. It can be either permanent or orthostatic. The Menard triad includes headaches, sweating, and palpitations and has a specificity of 94 % and a sensitivity of 91 %. These symptoms are most often paroxysmal and do not always occur concomitantly [2,8]. Acute cardiological complications may also be observed, mainly hypokinetic dilated cardiopathy with left or global heart failure [11]. Metabolic manifestations such as hyperglycemia or recent diabetes mellitus, lactic acidosis, and weight loss are also found in patients with PGL [12]. Other less specific signs may occur like anxiety, altered general condition, and emotional lability. Fever and flashes related to the co-secretion of multiple peptides (vasoactive intestinal peptide, P substance, and interleukin 6) are less frequently observed [8,12]. Manifestations related to the tumor syndrome such as vomiting, and pain depend on the location and size of the tumor. These data are consistent with our patient's case. Indeed, in our patient paroxysmal Menard triad was reported approximately 2 months before admission, and hypertensive peaks were noted during the echo endoscopy exploration, leading to the suspension of the procedure.

The diagnosis is performed by biology, which consists in confirming the hypersecretion of catecholamines, and imaging, which consists in identifying and locating the tumor and its mass effect on the adjuvant organs. Currently, the recommended tests are the determination of MN and NMN, which are inactive metabolites of catecholamines, either in plasma or in urine, positive testing is defined by a value of MN and/or NMN >4-fold upper normal limit of reference intervals times normal, with a sensitivity and specificity above 90 % [13,14]. In our case, the urinary catecholamines were positive with an MN value of 30-fold and an NMN value of the 23-fold upper normal limit of reference intervals.

Imaging allows for locating the tumor and assessing its local, regional, and remote extension. Usually, a CT scan shows a tissular image with an average size of 5 cm, with a spontaneous density of >10 HU, and with a significant elevation in the arterial phase, which gives the hypervascular character of PGLs. MRI is indicated in case of contraindication to CT scan, or in metastatic forms of PGL. In our case, we performed cervical and skull base MRI and an abdominal and pelvic CT scan showing a heterogeneous retroperitoneal 58 × 45 mm tumor without metastasis. Functional imaging is indicated if conventional imaging was not conclusive or if the measurement of catecholamines was doubtful. It can also be used to search for multifocal or secondary locations. Lenders et al. [15] and Bergeret et al. [16] proposed various nuclear imaging techniques depending on clinical presentation, location of the tumor, and genetic status. In the case of retroperitoneal paraganglioma, they recommend completing with 68Gallium scintigraphy, 18F-fluoro-2-deoxy-d-glucose positron emission tomography (18FDG-PET), or Fluorine-18-L-dihydroxyphenylalanine positron emission tomography (18FDOPA-PET) [17].

Surgery is the main treatment, preceded by α -blockers preparation that aims to achieve blood pressure control 2 weeks before surgery [2,18]. This medication helps to decrease systolic blood pressure below 130 mmHg and diastolic blood pressure below 80 mmHg and slows down the heart rate to around 70 bpm [15]. Doxazosin was the α-blocker used in our case. This particular preparation aims to prevent perioperative complications, chiefly severe hypertensive peaks caused by the mobilization of the tumor by the surgeon. If blood pressure is still not controlled on alpha-blockers, a calcium channel blocker such as Amlodipine, or a renin-angiotensin-aldosterone inhibitor may be added. Treatment with beta-blockers alone is contraindicated because of its exposure to the risk of hypertensive crisis. In our patient, blood pressure was controlled using only alpha-blockers. To control tachycardia after administration of α-blocker, a cardio-selective β-blocker should be preferred, and introduced in the coadministration of an α-adrenergic receptor blockers [19]. The preoperative diet must be equilibrated in sodium intake, with improved water intake, to prevent the risk of hypotension during the operation [15] Surgery must be performed by a team of experienced surgeons and anesthesiologists. The resection must be complete as in our case, in one piece. There is a risk of hemorrhage, due to the vascular nature of the tumor and the intimate relationship of some PGLs with large vessels. Open resection is usually preferred which will provide an exposure of the perirenal and infrarenal IVC, and laparoscopic resection is performed for small and noninvasive PGLs [20] [21]. The size of the tumor and its intimate adherence to the IVC led to open surgery in our patient. The risk of catecholaminergic discharge can induce severe hypertensive peaks or cardiac arrhythmia. The biopsy is therefore formally contraindicated, because of the risk of catecholaminergic discharge and the risk of hemorrhage [2]. In the case of vascular invasion, the reconstruction technique depends on the extension and location of the adjacent structures. However, there is also a risk of vein thrombosis, and as a consequence systemic heparinization is necessary, especially in patients with proof of partial vena cava thrombosis or deep vein thrombosis history [20]. In our patient, we used heparinized saline during the procedure. Reconstruction is performed by prosthetic materials such as PTFE [22]. For infrarenal IVC reconstruction, preservation of the IVC bifurcation is recommended because a supplementary bifurcated graft will be necessary. The vena cava bifurcation can be conserved, even if a portion of the common iliac vein is invaded, by performing the anastomosis with an incorporated patch repair of the common iliac vein. Distal anastomosis is achieved end-to-end under the renal veins [20]. In our case, a reconstruction of the IVC was performed using the PTFE, and the IVC bifurcation was preserved. To the best of our knowledge, this case of functional retroperitoneal paraganglioma invading the inferior vena cava, which is rare and fatal, has never been reported before in the existing literature.

Histological features show the hypervascular character, with chief cells and sustentacular cells arranged in clusters called Cell balls. Neuroendocrine markers are usually expressed by Chief cells in immunohistochemistry [5]. The same findings were reported in our case.

Diagnosis of malignancy of PGL is based on the presence of distant metastases in non-chromaffin tissues (liver, lung, bone, and lymph nodes) and is performed by imaging [23]. We excluded malignancy in our patient using a body scan.

PGLs are often associated with a germline mutation. The main incriminated genes are NF1 (involved in neurofibromatosis type 1), VHL (involved in Von Hippel Lindau syndrome), SDHB (involved in hereditary PGL syndrome), and RET (involved in multiple endocrine neoplasia type 2). The search for these mutations must be guided by the family history, the patient's age, the clinic, the biology, and the location of the tumor [24]. In our country, we didn't perform genetic testing.

Early follow-up includes blood pressure, heart rate, and blood glucose monitoring. In the early and long-term follow-up, biological and imaging monitoring is essential due to the risk of local or distant recurrence, and the development of distant metastasis [21]. In our patient, blood pressure and HbA1c were on the targets in the early and last follow-up. The current guidelines suggest that MN/NMN should be measured 2 to 6 weeks after surgery [25]. According to the French National Diagnostic and Care Protocol of the HAS, given the sensitivity of the MN/NMN measurements, there is no need for imaging if it is normal. However, if MN/NMN is elevated after surgery or if MN/NMN was not measured at the baseline, imaging should be performed 3 to 6 months after surgery.

## 4 Conclusion

Retroperitoneal PGL is a rare location of this type of tumor and causes a diagnostic challenge with malignant potential. In the elderly, this pathology is quite uncommon. This fragile population should be provided with special healthcare. Endocrinologists, surgeons, and anesthesiologists should work together to ensure an appropriate diagnosis and treatment of PGL, a suitable medical preparation, and the best conditions for a successful complete surgery.

## Ethical approval

This is a case report that does not require formal ethical committee approval. Data were anonymously registered in our database. Access to data was approved by the head of the department Hanane Latrech.

## Funding

There is no funding received for this work.

## CRediT authorship contribution statement

Mohamed Aymane Loukili and Imane Assarrar are the first co-authors, who wrote the manuscript.

Nada El Yamani and Anass Haloui participated in the writing of the manuscript.

Siham Rouf and Hanane Latrech participated in the writing, supervised and revised the final manuscript.

All authors approved the final version of the manuscript.

## Consent

Written informed consent was obtained from the patient for the publication of this case report and accompanying images. A copy of the written consent is available for review by the Editor-in-Chief of this journal on request.

## Guarantor

Hanane Latrech.

## Declaration of competing interest

The authors declare that they have no conflicts of interest.

## Data Availability

The patient data used to support the findings of this study can be retrieved from the archives of the Department of Endocrinology-Diabetology and Nutrition, at the Mohammed VI University Hospital of Oujda, Morocco.
